# Meta-Analysis of Clinical Efficacy and Safety of *Tripterygium wilfordii* Polyglycosides Tablets in the Treatment of Chronic Kidney Disease

**DOI:** 10.1155/2021/6640594

**Published:** 2021-05-13

**Authors:** Yan-Li Guo, Feng Gao, Tai-Wei Dong, Yang Bai, Qiao Liu, Ruo-Lan Li, Shu-Ting Yan, Mei Chen, Pei-Feng Wei, Miao-Miao Xi

**Affiliations:** ^1^Shaanxi University of Traditional Chinese Medicine, Shaanxi 712046, China; ^2^The Second Affiliated Hospital of Shaanxi University of Traditional Chinese Medicine, Shaanxi 712000, China; ^3^Affiliated Hospital of Shaanxi University of Chinese Medicine, Shaanxi 712000, China

## Abstract

**Objective:**

*Tripterygium wilfordii* polyglycosides tablet (TGt) is an oral preparation extracted from plant *Tripterygium wilfordii*. It has the effects of anti-inflammation and inhibition of cellular and humoral immunity. However, many reports of adverse reactions caused by TGt have limited its application. In this paper, the clinical efficacy and safety of TGt in the treatment of chronic kidney disease (CKD) were verified by data mining and analysis, so as to provide theoretical data support for the application and development of TGt.

**Methods:**

A computer search of the following databases was conducted: PubMed, Web of Science, CBM, VIP, Wanfang Data, and CNKI. The search time limit is from the establishment of the database to September 2020. We searched for clinical randomized controlled trials of TGt in the treatment of CKD. The main types of CKD involved are nephrotic syndrome (NS), primary nephrotic syndrome (PNS), refractory nephrotic syndrome (RNS), and IgA nephropathy (IgAN). RevMan 5.2 and Stata 12.0 software were used to evaluate the literature quality and analyze the data. Finally, GRADEpro software was used to evaluate the quality of evidence.

**Results:**

According to the inclusion and exclusion criteria, 75 articles with a total of 6418 subjects were included. The results of the meta-analysis showed that TGt could reduce 24-hour urinary protein, increase serum albumin, improve clinical efficacy, and reduce disease recurrence rate in patients (*P* < 0.05) with CKD compared with adrenocortical hormones or immunosuppressants. TGt could significantly reduce the level of serum creatinine (Scr) in patients with CKD (*P* < 0.05), but it was not significant in reducing the level of blood urea nitrogen (*P* > 0.05). In terms of safety evaluation, in patients with CKD, it could significantly reduce the incidence of gastrointestinal adverse reactions and neurogenic dizziness and headache (*P* < 0.05). However, in terms of adverse reactions such as liver injury, respiratory infection, and leukopenia, TGt was as harmful as corticosteroids or immunosuppressants (*P* < 0.05). The quality of the evidence was evaluated with GRADEpro software, and the results showed that TGt was strongly recommended for the treatment of CKD.

**Conclusion:**

TGt has certain efficacy in the treatment of CKD and has fewer side effects in certain types of diseases. The effect of TGt combined with other drugs is better than that of single use. This paper also has some limitations. Due to the limited number of the included studies, with all being from China, there may be methodological differences. Therefore, more high-quality literature data from different countries are needed.

## 1. Introduction

Kidney Disease: Improving Global Outcomes (KDIGO) organization defines chronic kidney disease (CKD) as abnormal kidney structure or function, which has an impact on health, lasting for 3 months [1]. Chronic kidney disease is a global public health problem, with a global prevalence rate of about 8–16% [2]. Growth disorders are common in patients with chronic kidney disease, and studies have found that there are significant changes in the axes of growth hormone, insulin-like growth factor-1, and insulin-like growth factor binding protein in these patients. The reason is tissue resistance to growth hormone rather than growth hormone deficiency [3]. Some studies have also found that insulin resistance is associated with the deterioration of renal function in patients with chronic kidney disease [4]. Significant changes in intestinal flora in patients with chronic kidney disease are considered to be an important factor leading to chronic inflammation. The study found that there were changes in the number and composition of intestinal bacteria in end-stage patients [5]. Anorexia is a common clinical manifestation in patients with chronic kidney disease, especially when renal function deteriorates. Some studies have shown that interleukin-6 and tumor necrosis factor-*α* were involved in appetite suppression in patients with chronic kidney disease. Leptin, a member of the interleukin-6 family, also plays a role in promoting inflammation in patients with chronic kidney disease [6, 7].

The prevalence of CKD in the United States rose from 13.8% in 2016 to 14.5% in 2017, and the number of cases of end-stage renal disease (ESRD) rose from 727912 in 2016 to 746557 in 2017. In 2017, the total health insurance expenditure for CKD and ESRD patients in the United States exceeded $120 billion, accounting for 7.2% of the total health insurance payments. According to the data of China Blood purification Case Information Registration System, by the end of 2018, there were about 580000 hemodialysis patients and 95000 peritoneal dialysis patients in China [8]. However, the main primary diseases of ESRD are primary glomerular disease, diabetic nephropathy, and so on. Nephrotic syndrome (NS) [9] shows a group of clinical symptoms of massive albuminuria, hypoproteinemia, high edema, and hyperlipidemia. NS can be divided into three categories: primary, secondary, and hereditary. Primary nephrotic syndrome (PNS) belongs to primary glomerular disease. The pathological type includes IgA nephropathy (IgAN), which accounts for about 30% of primary nephrotic syndrome. Secondary nephrotic syndrome is often caused by diabetic nephropathy (DN) and systemic lupus erythematosus. TGt is a fat-soluble mixture extracted from the root of *Tripterygium wilfordii*. It is the first anti-inflammatory and immunomodulatory Chinese herbal medicine studied and used in China, which is called “Chinese herbal hormone” [10]. TGt has the effects of anti-inflammation, immunosuppression or immune regulation, and antitumor. The scope of clinical application is very wide. Many studies have shown that TGt is effective in the treatment of many kinds of NS and RNS [11]. It can reduce the permeability of glomerular filtration membrane and reduce the excretion of urinary protein [12]. However, in recent years, the promotion of TGt has been seriously hindered by more and more reports of liver, kidney, and blood system damage caused by TGt. The purpose of this paper is to review a large amount of clinical data to verify the efficacy and safety of TGt in the treatment of CKD. It is expected to provide strong data support for the rational application of TGt.

## 2. Method

### 2.1. Data Retrieval

The literature comes from a published randomized controlled trial of TGt in the treatment of CKD. A computer search of the following databases was carried out: China National Knowledge Infrastructure (CNKI), VIP, Wanfang, Chinese Biomedical, Web of Science, Elsevier ScienceDirect, and Wiley Online Library. Both Chinese and English literature retrieval adopt the way of combining subject words with free words. The time of literature retrieval is from the establishment of the database to September 2020. The key words include “*Tripterygium wilfordii* polyglycoside tablets,” “*Tripterygium wilfordii* preparation,” “chronic kidney disease,” “IgA nephropathy,” “nephrotic syndrome,” “primary nephrotic syndrome,” “refractory nephrotic syndrome,” “diabetic nephropathy,” and “primary glomerular disease.”

### 2.2. Inclusion and Exclusion Criteria

The inclusion criteria are as follows: ① All randomized controlled trials evaluating TGt in English and Chinese were included. ② All the subjects were diagnosed with NS, PNS, RNS, and IgAN, regardless of sex, age, and race. ③ The experimental group was treated with TGt, which was not limited by dose and course of treatment. ④ The control group included basic routine treatment.

The exclusion criteria are as follows: ① the literature containing TGt in the control group was excluded. ② Animal experiments, conference papers, reviews, and so on were excluded. ③ Nonrandomized controlled trials were excluded.

### 2.3. Literature Screening, Data Extraction, and Quality Evaluation

Two researchers independently screened the literature, extracted the main data and information included, and cross-checked them. When screening the literature, the final inclusion of the literature is determined according to the order of reading title, abstract, and full text. The data information includes the name of the first author, the year of publication, the number of studies included, the number of effective treatments, intervention measures, course of treatment, outcome indicators, adverse reactions, type of trial design, random method, and blind method. If there are any differences, they shall be resolved through discussion or negotiation with the third author. If necessary, the author is consulted about the data indicators not mentioned in the literature. The quality of each article was evaluated according to the RCT bias risk assessment tool recommended by Cochrane.

### 2.4. Outcome Index

The clinical efficacy of TGt in the treatment of CKD included total clinical effective rate (total effective rate = (complete remission + partial remission)/total number × 100%), recurrence rate, 24-hour urinary protein, serum creatinine (Scr), blood urea nitrogen (BUN), and plasma albumin levels. According to the Chinese guidelines for evaluating the curative effect of glomerular disease [13], complete remission refers to the decrease of 24-hour urinary protein to ≤0.2 g/L, serum albumin ≥35 g/L, and the renal function being normal or improved. Partial remission refers to the decrease of 24-hour urine protein to 0.21–3.4 g/d, the relative baseline decrease of proteinuria by ≥50%, and the stability of renal function. Inefficacy refers to continuous treatment for 3 months without relief of symptoms or improvement or deterioration of urine protein test results. Recurrence refers to the occurrence of urinary protein ≥3.5 g/d after more than one month of remission. The main purpose of safety evaluation is to evaluate the incidence of adverse reactions. The adverse reactions are mainly manifested in the discomfort or harm to the digestive system, reproductive system, endocrine system, bone marrow and blood system, cardiovascular system, and nervous system [14].

### 2.5. Data Analysis

Statistical analysis was carried out using RevMan 5.3, provided by Cochrane Collaboration Network, and Stata 12.0 software. The second classification variable uses the odds ratio (OR) as the effect analysis statistic. The weighted mean difference (WMD) was used as the effect analysis statistic for the measurement data. All effects provide 95% confidence interval. The heterogeneity among the included results was analyzed by chi-square test (test level *α* = 0.05). At the same time, the heterogeneity was quantitatively judged by *I*^*2*^. When *P* ≥ 0.05 and *I*^2^ ≤ 50%, it is suggested that there is no obvious statistical heterogeneity, and the fixed effect model is used. When *P* < 0.05 and *I*^2^ > 50%, it is suggested that there is statistical heterogeneity, and metaregression is used to analyze the source of heterogeneity. If there is no obvious clinical heterogeneity, the random effect model is selected. According to the different intervention measures between the experimental group and the control group, Egger's linear regression method was used to test the existence of publication bias. The quality of each independent study was evaluated by using the Jadad scale, which is commonly used in the review of Cochrane system. Finally, GRADEpro software was used to evaluate the final evidence quality. A 2-sided *P* < 0.05 was considered statistically significant for all analyses.

## 3. Result

### 3.1. An Overview of the Inclusion of Literature

**Figure 1:**
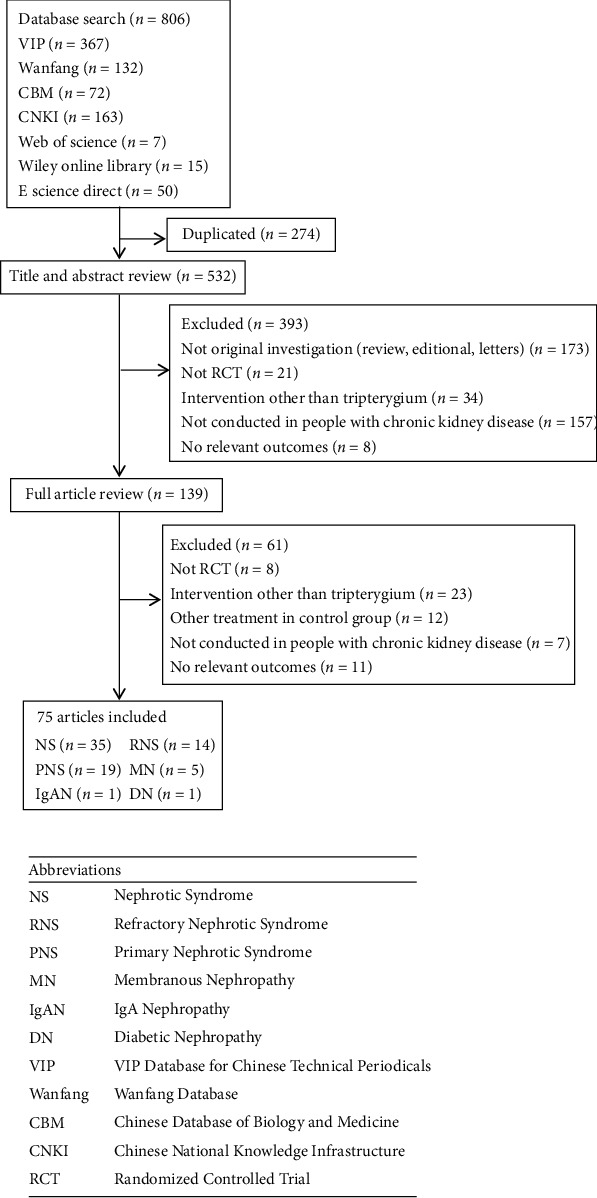
Results of literature screening.

**Figure 2:**
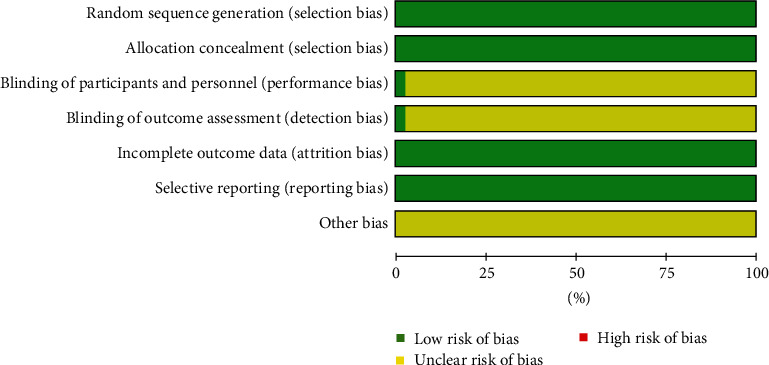
The literature is included in the quality evaluation map.

According to the retrieval strategy, a total of 806 related studies were found. According to the inclusion and exclusion criteria and one-by-one screening, we finally included 75 articles (Figure 1), with a total of 6418 subjects. The follow-up period was 1 month at least and 2 years at most. In all studies, the baseline characteristics of the experimental group and the control group were similar. Among the subjects, there were 2789 patients with NS, accounting for 43.46%. There were 1346 patients with RNS, accounting for 20.97%. There were 1879 patients with PNS, accounting for 29.28%. The rest were 291 patients with membranous nephropathy (MN), 64 patients with diabetic nephropathy, and 49 patients with IgA nephropathy. All the subjects were treated with basic routine treatment. The experimental group was treated with TGt alone or in combination with other drugs. The intervention measures in the control group were prednisone, prednisone acetate, methyl prednisone, Matimycophenolic acid (MMF), qiangdisong, leflunomitt (LEFT), or cyclophosphamide (CTX) (Table 1). According to the literature description, all trials are randomized controlled trials, but only 49 (62.8%) studies describe random sequence generation methods in detail. The quality results included in the overall literature are shown in Figure 2.

### 3.2. Clinical Effect

#### 3.2.1. Total Clinical Effective Rate

**Figure 3:**
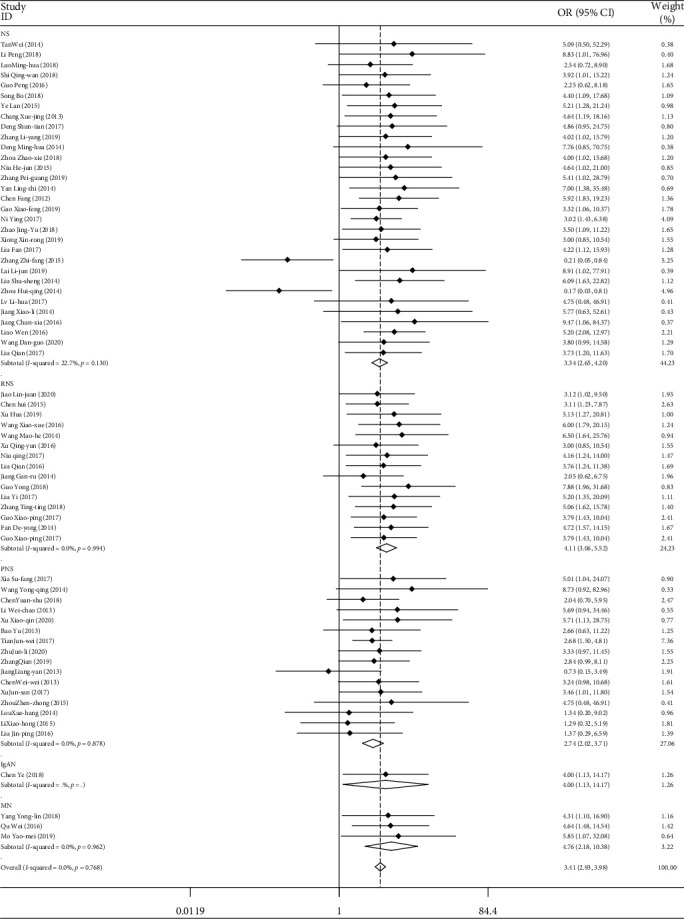
Comparison of clinical efficacy of different types of diseases between the experimental group and the control group.

A total of 66 studies included the total clinical effective rate of TGt in the treatment of CKD. A total of 5348 patients were involved. These studies were combined and analyzed according to the type of disease, the mode of medication, the course of treatment, and the age of the patients. The weighted combination results (Table 2, Figure 3) showed that the data of the 66 studies were homogeneous (*I*^2^ = 0.0%, *P* > 0.05). The combined effect OR was modeled by fixed effect, the OR = 3.415, and the 95% confidence interval was (2.933, 3.975). For the test of comprehensive OR, the difference was statistically significant (*z* = 15.84, *P* < 0.05). Therefore, we think that TGt is more effective than other drugs in the treatment of chronic kidney disease. Publication bias was detected by Egger's linear regression (*P* < 0.05). The results of metaregression analysis showed that different types of diseases, mode of medication, courses of treatment, and age of patients had influence on the combined effect (*P* < 0.05). Although the pathogenesis of each disease is crossed, it is also different. For example, the treatment of diabetic nephropathy with *Tripterygium wilfordii* polyglycosides may be related to increasing the activity of catalase (CAT) in serum and glutathione peroxidase (GSH-Px) in kidney tissue, reducing the content of malondialdehyde (MDA) in kidney tissue and the level of superoxide anion (O2 -) in serum, inhibiting oxidative stress, and enhancing the ability of antioxidation [86, 87]. The four aspects of appetite, hormone level, inflammatory state, and metabolic function of patients with chronic kidney disease influence and interact with each other [88]. Therefore, different body factors will have a certain impact on the efficacy of medication.

#### 3.2.2. Recurrence Rate

**Figure 4:**
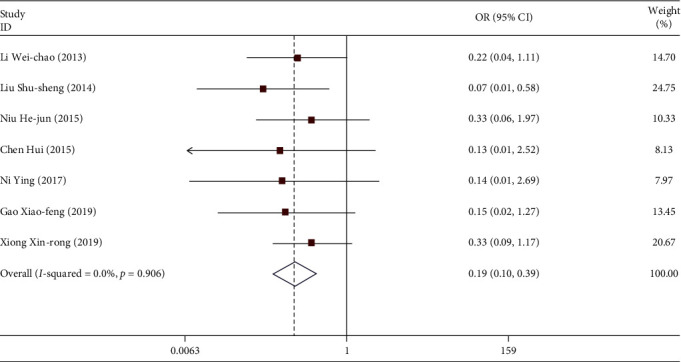
Comparison of recurrence between the experimental group and control group.

A total of 7 studies included the recurrence of the disease in the treatment of CKD. A total of 536 patients were enrolled. Among them, 65 patients relapsed. The weighted merging results (Figure 4) showed that the data of the 7 studies were homogeneous (*I*^2^ = 0.0%, *P* > 0.05). The fixed effect model was used, the combined OR = 0.194, and the 95% confidence interval was (0.097, 0.388). For the test of comprehensive OR, *z* = 4.64, *P* < 0.05. There are statistical differences. In patients treated with TGt, the recurrence rate can be reduced compared with the control group. There was no publication bias in the Egger linear regression test (*P* > 0.05).

#### 3.2.3. 24-Hour Urinary Protein

**Figure 5:**
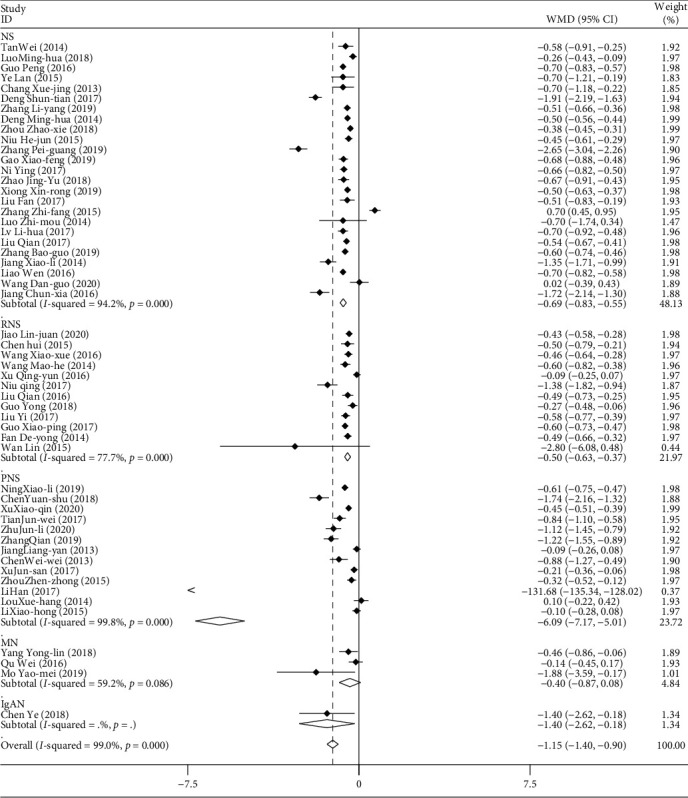
Comparison of 24-hour urinary protein in patients with different types of diseases between the experimental group and the control group.

A total of 54 studies included 24-hour urinary protein data, with a total of 4495 patients. The combination and subgroup analysis of 24 h urinary protein volume were performed according to disease type, medication method, course of treatment, and patient age. The results of the meta-analysis showed that the results of 8 studies were not statistically significant. The results of weighted combination (Table 3, Figure 5) showed that the data of the 54 studies had great heterogeneity (*I*^2^ = 99.0%, *P* < 0.05). The random effect model was used in the combined effect. WMD = -0.503 and the 95% confidence interval was (−0.526, −0.481). For the comprehensive WMD test, *z* = 43.52, *P* < 0.05. The difference is statistically significant. Therefore, it can be considered that the ability of TGt to reduce 24-hour urinary protein is higher than that of other therapeutic agents. Publication bias was detected by Egger's linear regression (*P* < 0.05). The results of metaregression analysis showed that different types of diseases, mode of medication, courses of treatment, and age of patients had no effect on the combined effect (*P* > 0.05). However, subgroup analysis showed that TGt was more effective in the treatment of PNS than other disease types. The effect of reducing 24-hour urinary protein in the elderly (>66 years old) was better than that in the young patients treated with TGt. The effect of combined treatment was better than that of TGt alone in reducing 24-hour urinary protein.

#### 3.2.4. Serum Creatinine (Scr)

**Figure 6:**
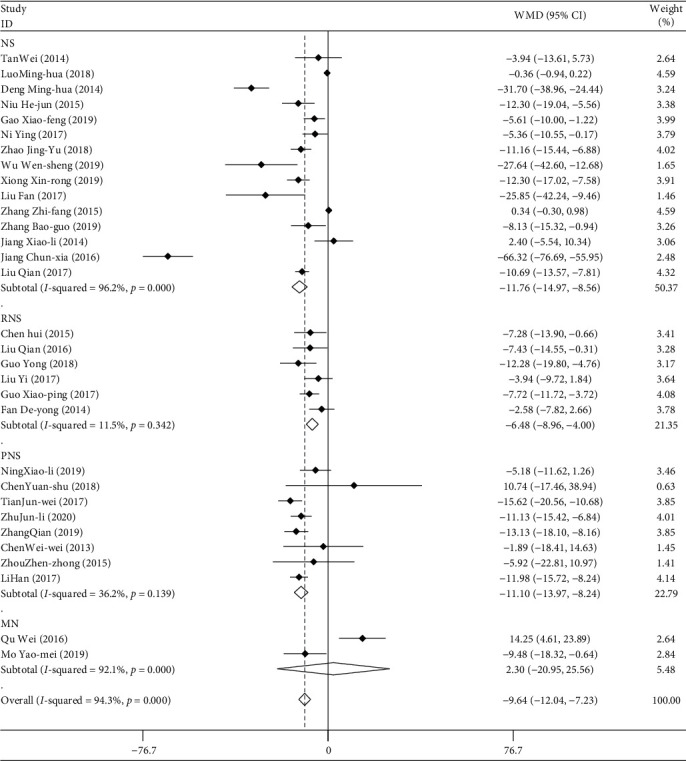
Comparison of serum creatinine level of drug intervention between the experimental group and control group with different types of diseases.

A total of 31 studies included data on Scr for a total of 2580 patients. The Scr data were combined and subgroup-analyzed according to the type of disease, the mode of medication, the course of treatment, and the age of the patients. The results of weighted combination (Table 4) show that the data of the 31 studies have great heterogeneity (*I*^2^ = 94.3%, *P* < 0.05). The random effect model was used in the combined effect. WMD = −9.636, and the 95% confidence interval was (−12.039, −7.233). For the comprehensive WMD test, *z* = 7.86, *P* < 0.05. The difference is statistically significant. Therefore, it can be considered that TGt has higher ability to reduce Scr than other therapeutic agents. Publication bias was detected by Egger's linear regression (*P* < 0.05). The results of metaregression analysis showed that different types of diseases and courses of treatment had influence on the combined effect (*P* < 0.05). The results of subgroup analysis showed that TGt had no statistical significance in reducing Scr in patients with MN and DN compared with the control group (Figure 6). As for the course of treatment, when it is greater than or equal to 12 months, there is no statistical significance compared with the control group.

#### 3.2.5. Blood Urea Nitrogen (BUN)

**Figure 7:**
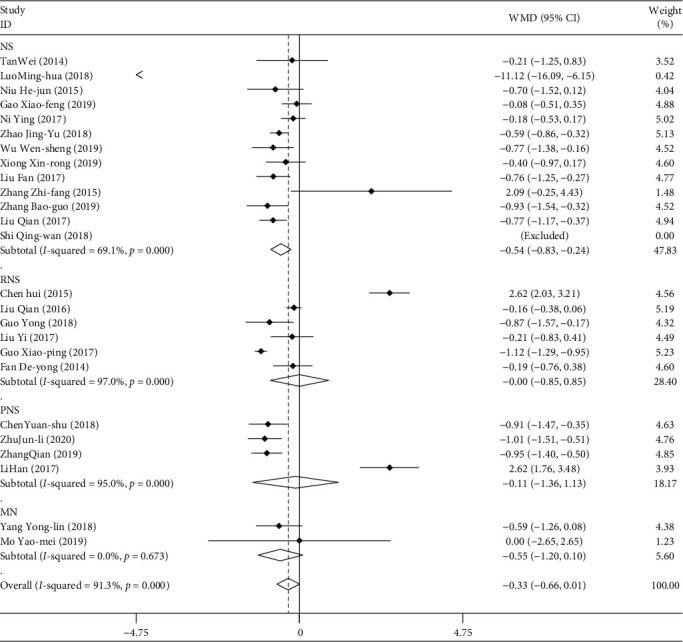
Comparison of drug intervention on blood urea nitrogen between the experimental group and control group of different types of diseases.

A total of 24 studies included BUN data for a total of 2057 patients. The data of BUN were combined and analyzed according to the type of disease, the mode of medication, the course of treatment, and the age of the patients. The results of weighted combination (Table 5, Figure 7) showed that the data of the 24 studies had great heterogeneity (*I*^2^ = 91.3%, *P* < 0.05). The random effect model was used in the combined effect. WMD = −0.326, and the 95% confidence interval was (−0.661, 0.009). For the comprehensive WMD test, *z* = 1.91, *P* > 0.05. There is no statistical difference. That is, the ability of TGt to reduce BUN level is similar to that of other therapeutic agents. There was no publication bias in the Egger linear regression test (*P* > 0.05). The results of metaregression analysis showed that the course of treatment had an effect on the combined effect (*P* < 0.05). The results of subgroup analysis showed that TGt had statistical significance in the treatment of NS compared with the control group (*P* < 0.05). Regarding the course of treatment, when the treatment time was ≥12 months, it was statistically significant compared with the control group. That is, when the treatment time of TGt is more than 12 months, the level of BUN can be significantly reduced.

#### 3.2.6. Serum Albumin

**Figure 8:**
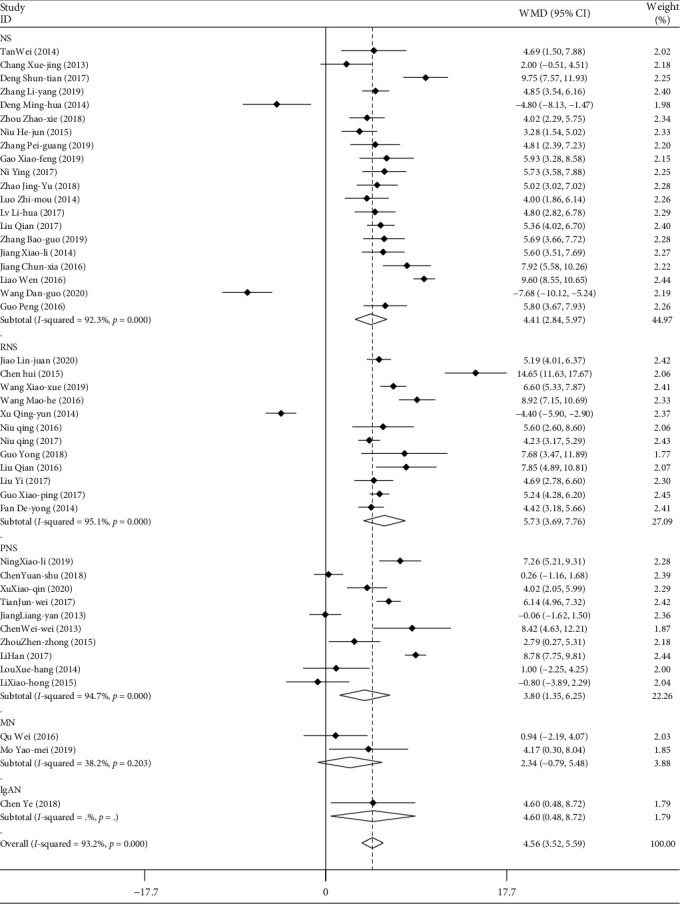
Comparison of drug intervention on serum albumin between the experimental group and the control group with different types of diseases.

A total of 45 studies included serum albumin data for a total of 3550 patients. The serum albumin data were combined and subgroup-analyzed according to the type of disease, the mode of medication, the course of treatment, and the age of the patients. The results of weighted combination (Table 6, Figure 8) showed that the data of the 45 studies had great heterogeneity (*I*^2^ = 93.2%, *P* < 0.05). The random effect model was used in the combined effect. WMD = 4.557, and the 95% confidence interval was (3.523, 5.529). For the comprehensive WMD test, *z* = 8.64, *P* < 0.05. There are statistical differences. That is, the ability of TGt to increase serum albumin level is higher than that of other therapeutic agents. There was no publication bias detected by Egger's linear regression (*P* > 0.05). The results of metaregression analysis showed that different types of diseases, mode of medication, courses of treatment, and age of patients had influence on the combined effect (*P* < 0.05).

### 3.3. Safety Evaluation

#### 3.3.1. Total Incidence of Adverse Reactions

**Figure 9:**
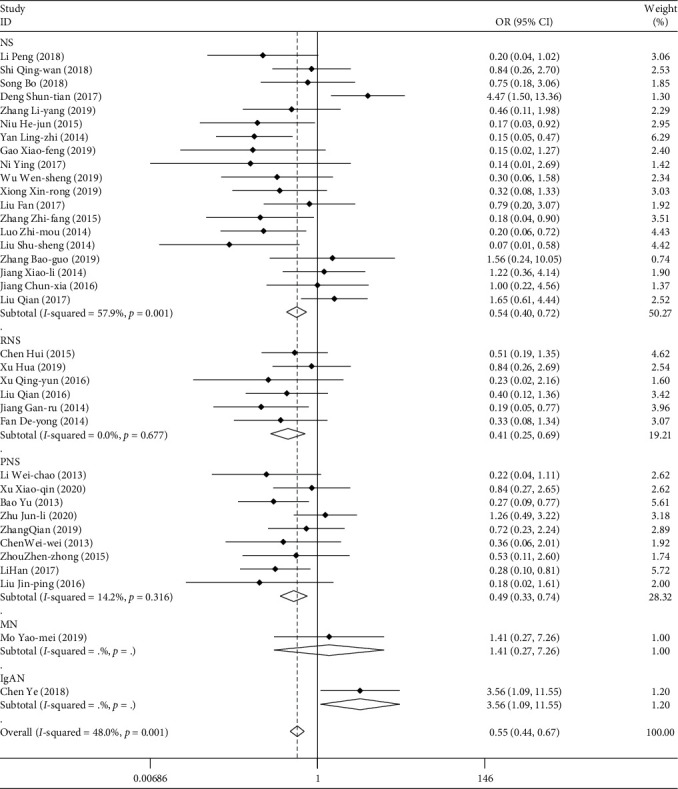
Comparison of adverse reactions caused by drugs in different types of experimental group and control group.

A total of 36 studies reported the following 12 adverse reactions: gastrointestinal adverse reactions (30), leukopenia (18), dizziness and headache (9), thrombocytopenia (2), liver injury (11), respiratory tract infection (12), elevated blood glucose (4), menstrual disorder (3), elevated blood pressure (2), elevated glutamic pyruvic transaminase (4), other adverse reactions (palpitation, insomnia, hair loss, etc.). A total of 2614 patients were included in the study, and a total of 482 cases of adverse reactions occurred. These studies were combined and analyzed according to the type of disease, the mode of medication, the course of treatment, and the age of the patients. The weighted merging results (Table 7, Figure 9) show that the data of the 36 studies are homogeneous (*I*^2^ = 48%, *P* < 0.05). Using the fixed effect model, the combined OR = 0.546, and the 95% confidence interval was (0.443, −0.673). For the comprehensive OR test, *z* = 5.68, *P* < 0.05. There are statistical differences. Patients treated with TGt were less likely to have adverse events than those treated with other drugs. Publication bias was detected by Egger's linear regression (*P* < 0.05). The results of metaregression analysis showed that the course of treatment and the age of the patients had influence on the combined effect (*P* < 0.05). The results of subgroup analysis showed that TGt combined with other drugs could reduce the incidence of adverse reaction events, especially for older patients.

#### 3.3.2. Gastrointestinal Adverse Reactions

**Figure 10:**
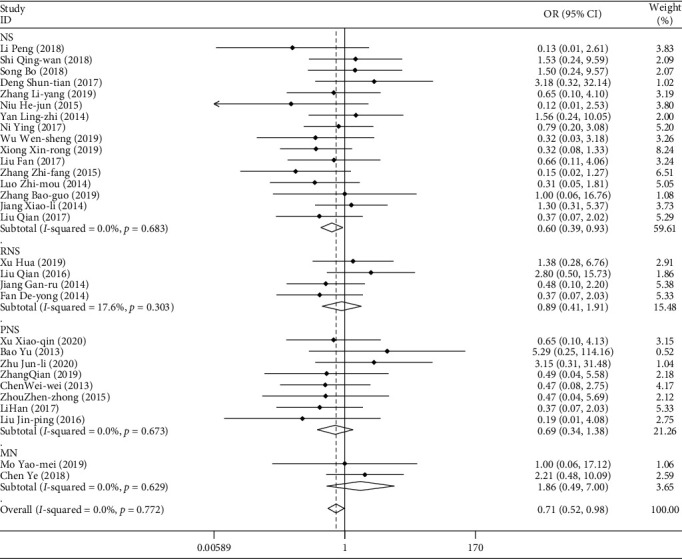
Comparison of gastrointestinal adverse reactions induced by drugs in different types of diseases between the experimental group and the control group.

A total of 2190 cases of gastrointestinal adverse reactions (nausea, vomiting, etc.) occurred in patients treated with TGt The data were combined, and their subgroups were analyzed according to the type of disease, the mode of medication, the course of treatment, and the age of the patients. The weighted merging results (Table 8, Figure 10) show that the data of the 30 studies are homogeneous (*I*^2^ = 0.0%, *P* > 0.05). Using the fixed effect model, the combined OR = 0.711, and the 95% confidence interval is (0.517, 0.980). For the comprehensive OR test, *z* = 2.09, *P* < 0.05.

There are statistical differences. The probability of gastrointestinal adverse events in patients treated with TGt was lower than that in the control group. There was no publication bias in the Egger linear regression test (*P* > 0.05). The results of metaregression analysis showed that the course of treatment and the age of the patients had influence on the combined effect (*P* < 0.05). The results of subgroup analysis showed that the probability of gastrointestinal adverse events in patients with NS treated with TGt was lower than that in patients with other diseases.

#### 3.3.3. Leukopenia

**Figure 11:**
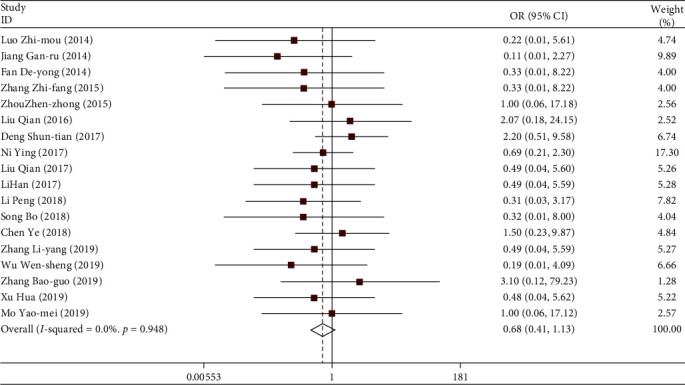
Comparison of the intervention of drugs on leukopenia between the experimental group and the control group.

A total of 18 studies included leukopenia in patients. A total of 1248 patients were involved. Among them, 59 patients had leukopenia in their blood. The weighted combination results (Figure 11) show that the data of the 18 studies are homogeneous (*I*^2^ = 0.0%, *P* > 0.05). Using the fixed effect model, the combined OR = 0.68, and the 95% confidence interval was (0.41, 1.13). For the comprehensive OR test, *z* = 1.48, *P* > 0.05. There is no statistical difference. Patients treated with TGt were as likely to have leukopenia as those in the control group. There was no publication bias detected by Egger's linear regression (*P* > 0.05).

#### 3.3.4. Liver Injury

**Figure 12:**
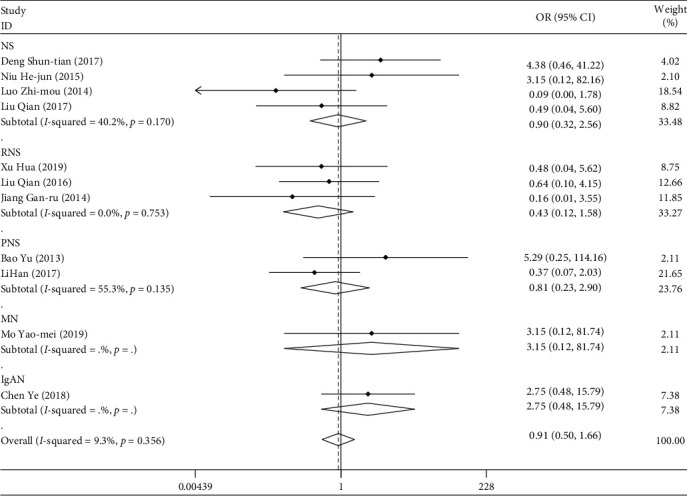
Comparison of liver injury induced by drugs in the experimental group and the control group.

A total of 11 studies included drug-induced liver injury. A total of 661 patients were enrolled. Among them, 39 patients had liver injury. The weighted merging results (Figure 12) showed that the data of the 11 studies were homogeneous (*I*^2^ = 9.3%, *P* > 0.05). Using the fixed effect model, the combined OR = 0.906, and the 95% confidence interval was (0.495, 1.658). For the comprehensive OR test, *z* = 0.32, *P* > 0.05. There is no statistical difference. Patients treated with TGt are as likely to have liver injury events as other drugs. There was no publication bias in the Egger linear regression test (*P* > 0.05).

#### 3.3.5. Respiratory Tract Infection

**Figure 13:**
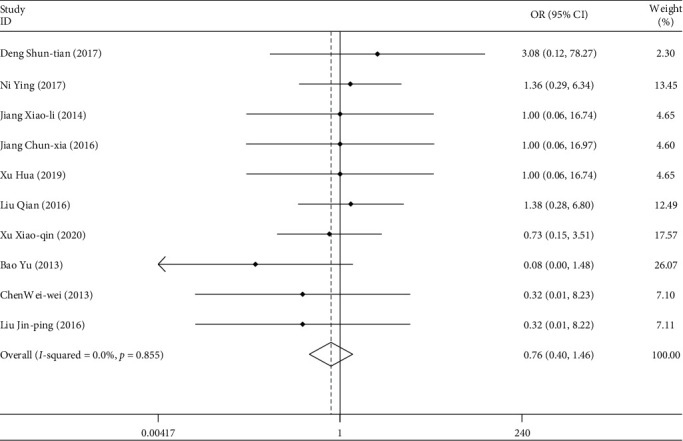
Comparison of respiratory tract infection caused by drugs in the experimental group and the control group.

A total of 706 patients were included in 10 studies. Among them, 35 patients developed respiratory tract infection. The weighted merging results (Figure 13) showed that the data of the 10 studies were homogeneous (*I*^2^ = 0.0%, *P* > 0.05). The fixed effect model was used, the combined OR = 0.76, and the 95% confidence interval was (0.395, 1.46). For the comprehensive OR test, *z* = 0.82, *P* > 0.05. There is no statistical difference. Patients treated with TGt are as likely to develop respiratory infections as other drugs. There was no publication bias in the Egger linear regression test (*P* > 0.05).

#### 3.3.6. Dizziness and Headache

**Figure 14:**
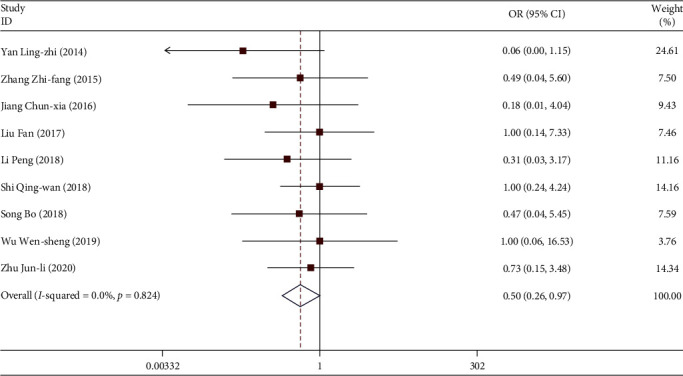
Comparison of dizziness and headache caused by drugs in the experimental group and the control group.

A total of 719 patients were included in 9 studies. Among them, 39 patients developed dizziness and headache. The incidence of adverse events and its subgroups were analyzed according to the type of disease, the course of treatment, and the age of the patients. The weighted combination results (Figure 14) show that the 9 studies are homogeneous (I^2^ = 0.0%, *P* > 0.05). Using the fixed effect model, the combined OR = 0.498, and the 95% confidence interval is (0.257, 0.965). For the comprehensive OR test, *z* = 2.06, *P* < 0.05. There are statistical differences. Patients treated with TGt were less likely to have dizziness and headache than those treated with other drugs. Publication bias was detected by Egger's linear regression (*P* < 0.05). The results of metaregression analysis showed that the type of disease, the course of treatment, and the age of the patients had no influence on the combined effect (*P* > 0.05). The results of subgroup analysis showed that the incidence of dizziness and headache in patients with NS treated with TGt was lower than that in patients with other diseases (*P* < 0.05). The incidence of dizziness and headache in middle-aged patients treated with TGt was lower than that in the elderly (*P* < 0.05).

#### 3.3.7. Elevated Blood Sugar

**Figure 15:**
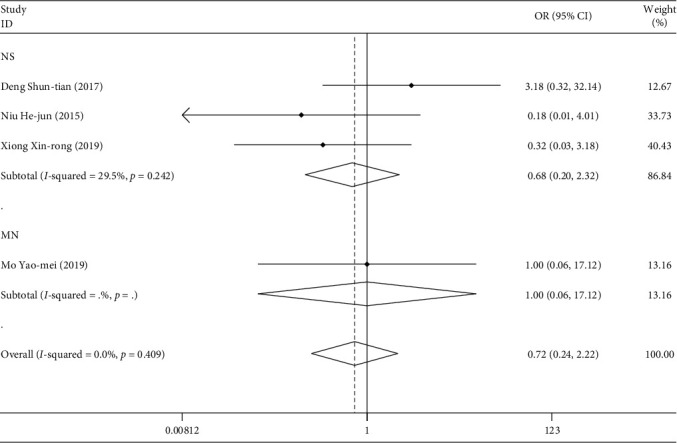
Comparison of the increase of blood glucose induced by drugs in the experimental group and the control group.

A total of 213 patients were included in 4 studies. Among them, 12 patients had elevated blood glucose. The results of weighted combination show that the data of the 4 studies are homogeneous (*I*^2^ = 0.0%, *P* > 0.05). Using the fixed effect model, the combined OR = 0.724, and the 95% confidence interval was (0.235, 2.225). For the comprehensive OR test, *z* = 0.56, *P* > 0.05 (Figure 15). There is no statistical difference. Patients treated with TGt are as likely to have elevated blood sugar as those treated with other drugs. There was no publication bias in the Egger linear regression test (*P* > 0.05).

### 3.4. Outcome Indicators' Evidence Quality Rating

**Figure 16:**
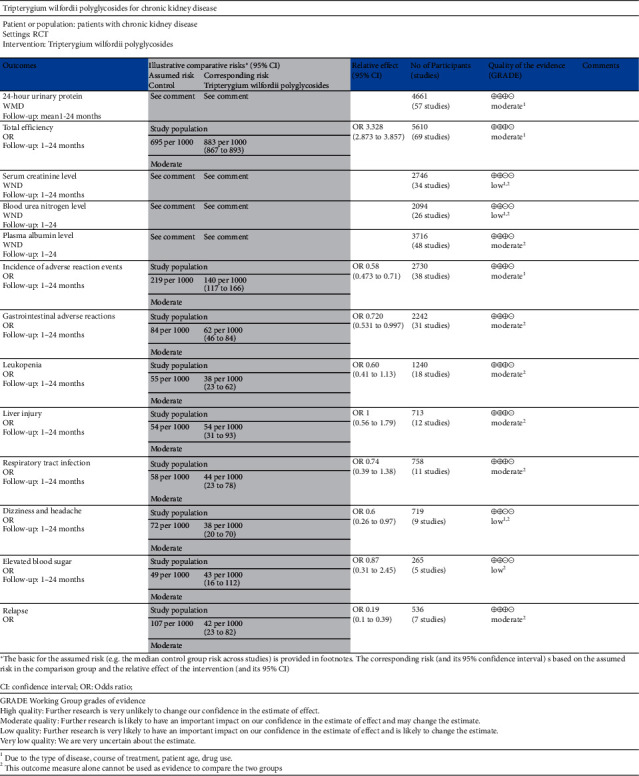
Evaluation of evidence quality of outcome indicators of TGt in the treatment of chronic nephropathy.

The quality of evidence was evaluated by GRADEpro software, and the results showed that TGt is recommended to treat chronic nephropathy. See Figure 16.

## 4. Discussion

Chronic kidney disease is a clinical syndrome gradually developed from a variety of primary and/or secondary kidney diseases in the acute stage, which may seriously endanger human life and health. The pathogenesis of the disease is complex; the disease often develops continuously and eventually develops into end-stage renal failure [89]. TGt is a highly polar mixture of fat-soluble components extracted and purified from the roots of *Tripterygium wilfordii*, including diterpene lactones, alkaloids, and triterpenes [90]. Some studies have shown that TGt cannot replace hormones but can be used as an effective adjuvant drug [91]. Clinically, in the treatment of renal disease, it can effectively protect the integrity of the charge barrier of glomerular filtration membrane, significantly reduce renal albuminuria, and improve nephropathy [92] [93]. Studies have shown that TGt can cause ovarian damage and menstrual disorders in women, while it may lead to infertility in men. Other studies have shown that TGt may cause myelosuppression [94]. In this paper, the clinical effective rate was evaluated according to the Chinese guidelines on the efficacy of glomerular diseases. Compared with adrenocortical hormones or immunosuppressants, TGt can significantly reduce 24-hour urinary protein, increase plasma albumin, and improve the cure rate in patients with chronic kidney disease. According to the results of meta-analysis, compared with adrenocortical hormones or immunosuppressants, TGt can be used for treating patients with RNS and MN. In different types of chronic renal disease, TGt was used to reduce 24-hour urinary protein in patients with primary nephrotic syndrome. The results of subgroup analysis showed that the effect of combination was better than that of TGt alone. Patients need to take medication according to the symptoms and follow the doctor's advice; not the longer the medication, the better the effect. Only 7 articles included the data of disease recurrence. According to statistical analysis, TGt can significantly reduce the recurrence rate of patients with chronic kidney disease. However, compared with adrenocortical hormones or immunosuppressants, TGt can significantly reduce the level of Scr in patients with chronic kidney disease, but the level of blood urea nitrogen is not significant. Therefore, the use of TGt for the recovery of renal function of patients needs the examination a lot of data. In evaluating the safety of TGt, the results of meta-analysis showed that TGt could significantly reduce the incidence of neurogenic dizziness and headache and gastrointestinal adverse reactions compared with adrenocortical hormones or immunosuppressants. However, the effects on liver injury, respiratory infections, and hematological adverse reactions such as leukopenia were similar to those of adrenocortical hormones or immunosuppressants, and there was no statistical difference. A total of 75 articles were included in this paper, of which 38 articles recorded adverse reactions caused by drugs. However, only 2 studies [31, 44] reported menstrual disorder, and the patients in these two articles were all patients with NS, but there were no such reports in patients with other disease types. This is different from the research conclusion of ZhuBin scholars. Therefore, more high-quality literature is needed to further explore its adverse reactions, especially for different types of chronic kidney disease and other diseases.

## Figures and Tables

**Table 1:** Basic information and quality evaluation results included in the literature.

Author	Year	Disease	Included in the study			Follow-up time (mo)	Course of disease (y)	Intervention measures	
			*T*/*C*	M/F	Age (y)			*T*	*C*
Tan Wei [15]	2014	NS	15/15	20/10	28.5 ± 3.5	6–12	1.13	TG	Glucocorticoid
Li Peng [16]	2018	NS	30/30	33/27	48.62 ± 1.41/48.63 ± 11.25	6	1.52 ± 1.41/1.65 ± 1.47	TG	Prednisone
Luo Minghua [17]	2018	NS	48/48	56/40	51.6 ± 10.3/51.2 ± 10.1	6	5.1 ± 1.9/4.9 ± 1.7	TG	Prednisone
Shi Qingwan [18]	2018	NS	50/50	—	49 ± 6.9/47 ± 5.7	6	19 ± 6.7/18 ± 5.7	TG	Prednisone acetate
Guo Peng [19]	2016	NS	40/40	43/37	45.7 ± 2.1/45.5 ± 3	3	8.2 ± 1/7.5 ± 1.2	TG	CTX
Song Bo [20]	2018	NS	36/35	38/33	50.26 ± 9.27/48.68 ± 9.38	6	14.53 ± 6.35/13.28 ± 6.13	TG	Prednisone
Ye Lan [21]	2015	NS	30/30	42/18	33.9 ± 3.8	18–24	—	TG	Methylprednisolone + dipyridamole
Chang Xuejing [22]	2013	NS	41/41	66/22	34.9 ± 3.9	18–24	—	TG	Methylprednisolone + dipyridamole
Deng Shuntian [23]	2017	NS	36/36	43/29	48.2 ± 2.5/47.6 ± 2.4	8	3.9 ± 1.9/3.8 ± 1.2	TG	Prednisone acetate + CTX
Jiang Xiaoli [24]	2014	NS	31/31	38/24	66.7 ± 4.2/64.3 ± 4.5	6	8 ± 4.1/8.7 ± 4.5	TG	Methylprednisolone
Jiang Chunxia [25]	2016	NS	24/24	27/21	56.07 ± 6.97/54.27 ± 6.26	—	—	TG	Methylprednisolone
LiaoWen [26]	2016	NS	56/56	57/55	70.2 ± 5.6/70.5 ± 5.4	6	0.8 ± 0.38/0.79 ± 0.4	TG	Methylprednisolone
Wan Danguo [27]	2020	NS	60/60	36/24	69.43 ± 3.74	6	3.52 ± 0.94	TG	Methylprednisolone
Zhang Liyang [28]	2019	NS	44/44	47/41	41.11 ± 7.24/41.05 ± 7.05	12	3.18 ± 1.08/3.25 ± 1.02	TG	Methylqiangdisong
Deng Minghua [29]	2014	NS	23/23	21/25	31.6 ± 6.9/35.2 ± 8.7	2	—	TG	Sufficient prednisone
Zhou Zhaoxie [30]	2018	NS	45/45	50/40	52.5 ± 6.5/52.3 ± 6.3	12	3.2 ± 1.1/3.2 ± 1	TG	Prednisone
Niu Hejun [31]	2015	NS	20/20	24/16	62.71 ± 9.13	12	0.25–10	TG	Prednisone
Zhang Peiguang [32]	2019	NS	25/25	28/22	39.87 ± 5.21/39.94 ± 5.23	6	1.57 ± 0.22	TG	Basics + prednisone acetate
Yan Lingzhi [33]	2014	NS	30/30	38/22	45.2 ± 18.3/43.8 ± 14.3	3	10.8 ± 3.5/6.9 ± 3.1	TG	CTX
Chen Fang [34]	2012	NS	50/50	46/54	23–66	3–6	0.5–12	TG	CTX
Liu Qian [35]	2017	NS	41/41	53/29	39.5 ± 8.6/38.9 ± 8.5	6	2.9 ± 1.1/2.8 ± 1.3	TG	Prednisone
Gao Xiaofeng [36]	2019	NS	42/42	34/50	36.74 ± 6.28/37.18 ± 6.94	6	0.94 ± 0.39/1.01 ± 0.44	TG	Basics + MMF
Ni Ying [37]	2017	NS	60/60	51/69	40.2 ± 8.3/38.5 ± 7.3	6	1.12 ± 0.71/1.05 ± 0.68	TG	MMF
Zhao Jingyu [38]	2018	NS	36/36	39/33	37.77 ± 5.42/37.68 ± 5.35	6	1.61 ± 0.27/1.58 ± 0.24	TG	Basic + prednisone acetate
Wu Wensheng [39]	2019	NS	42/42	41/43	51.06 ± 4.18/50.28 ± 4.12	6	1.64 ± 0.86/1.56 ± 0.85	TG	Regular + prednisone
Xiong Xinrong [40]	2019	NS	40/40	45/35	64.1 ± 3.5/63.2 ± 4.2	3–6	5.1 ± 1.5/5.2 ± 1.3	TG	Prednisone
Liu Fan [41]	2017	NS	64/64	73/55	52.39 ± 12.37/51.82 ± 13.86	6	1.62 ± 0.68/1.55 ± 0.69	TG	Prednisone
Zhang Zhifang [42]	2015	NS	40/40	49/31	51.02 ± 9.27/49.16 ± 7.28	6	1.25 ± 1.24/1.34 ± 1.29	TG	Prednisone
Lai Lijun [43]	2019	NS	29/29	36/22	50.45 ± 1.83/49.12 ± 1.97	3	1.27 ± 0.11/1.35 ± 1.02	TG	Prednisone
Luo Zhimou [44]	2014	NS	44/30	—	47.28 ± 3.17/47.28 ± 3.17	6	3.4 ± 0.3	TG	CTX
Liu Shusheng [45]	2014	NS	50/50	53/47	1.5–14/2–13	12	—	TG	Hormone
Zhou Huiqing [46]	2014	NS	50/50	56/44	34.3 ± 1.2	12–18	2.1 ± 0.1	TG	Prednisone
Lv Lihua [47]	2017	NS	20/20	24/16	63.14 ± 9.07/62.32 ± 8.08	12	6.2 ± 1.4/5.3 ± 1.8	TG	Prednisone
Zhang Baoguo [48]	2019	NS	30/30	37/23	51.02 ± 5.84/51.06 ± 5.99	12	—	TG	Prednisone
Liu Yi [49]	2017	RNS	42/42	48/36	36.72 ± 5.41/37.12 ± 5.83	12	3.26 ± 1.18/3.45 ± 1.27	TG	Prednisone
Zhang Tingting [50]	2018	RNS	31/31	34/28	54.21 ± 3.74/55.09 ± 3.48	3	—	LEFT + TG	CTX + prednisone
Liu Qian [51]	2016	RNS	30/30	25/35	36.9 ± 4.3/36.6 ± 4.2	6	9.9 ± 3.1/9.5 ± 3.6	TG + MMF	Routine + CTX + hormone
Niu Qing [52]	2017	RNS	45/45	47/43	63.25 ± 3.21/64.25 ± 4.23	3	8.2 ± 3.2/8.6 ± 3	TG	CTX
Guo Yong [53]	2018	RNS	30/30	33/27	56.4 ± 11.2/57.1 ± 12.3	6	3.7 ± 1.1/3.6 ± 1.2	LEFT + TG	CTX + prednisone
Guo Xiaoping [54]	2017	RNS	85/85	88/82	35.19 ± 5.98/36.54 ± 6.07	4	1.01 ± 0.23/0.92 ± 0.22	TG	MMF
Wan Lin [55]	2015	RNS	104/84	122/65	51.8 ± 12.2/45.3 ± 11.8	1	—	TG	Dexamethasone
Jiao Linjuan [56]	2020	RNS	53/53	57/49	36.19 ± 4.82/35.48 ± 4.5	12	3.17 ± 0.42/3.24 ± 1.06	TG	Prednisone acetate
Chen Hui [57]	2015	RNS	42/40	36/46	34.7 ± 6.2/35.1 ± 6.7	6	0.82 ± 0.38/0.85 ± 0.4	TG	Routine + prednisone + MMF
Xu Hua [58]	2019	RNS	31/31	51/11	39.68 ± 3.27/35.97 ± 3.45	6	0.83 ± 0.11/0.83 ± 0.12	TG	MMF
Wang Xiaoxue [59]	2016	RNS	40/40	47/33	34.9 ± 7.6/35.6 ± 6.4	3	3.5 ± 1.4/3.7 ± 1.3	TG	Prednisone
Wang Maohe [60]	2014	RNS	33/33	36/30	35.1 ± 2.7/34.6 ± 2.4	12	3.5 ± 2.1/3.6 ± 1.9	TG	Prednisone
Xu Qingyun [61]	2016	RNS	40/40	47/33	39.3 ± 27.9/45.1 ± 36.9	2	6.2 ± 5.9/5.8 ± 4.7	TG	Glucocorticoid
Fan Deyong [62]	2014	RNS	48/48	53/43	34.6 ± 7.4/35.2 ± 7.8	12	3.2 ± 1.2/3.3 ± 1.3	TG	Basic + prednisone
Xia Sufang [63]	2017	PNS	71/71	79/63	76.5 ± 3.2/72.6 ± 2.3	6	—	TG	Prednisone
Wang Yongqing [64]	2014	PNS	17/17	18/16	51.12 ± 10.39	2	—	TG	Prednisone
Li Han [65]	2017	PNS	47/47	46/48	68.21 ± 1.79/67.45 ± 1.54	2	0.83 ± 0.089/0.82 ± 0.094	TG	Prednisone
Jiang Ganru [66]	2014	PNS	32/28	34/26	56.4 ± 12/55.4 ± 11.2	6	2.13 ± 0.42/2.21 ± 0.38	LEFT + TG + prednisone	CTX + prednisone
Bao Yu [67]	2013	PNS	36/36	48/24	69.2 ± 8.06	>6	0.74 ± 0.45	TG	Prednisone
Tian Junwei [68]	2017	PNS	140/140	151/129	72.19 ± 9.45/71.34 ± 9.28	2	2.92 ± 1.12/2.87 ± 1.15	TG	Prednisone + CTX
Lou Xuehang [69]	2014	PNS	19/22	24/17	60.6 ± 1.6/62.1 ± 5.8	12	—	TG + hormone	CTX + hormone
Zhu Junli [70]	2020	PNS	44/44	47/41	71.96 ± 8.63	3	1.99 ± 0.96	TG	Regular + benazepril
Zhang Qian [71]	2019	PNS	51/51	48/54	71.02 ± 9.44/72.13 ± 8.76	2	2.47 ± 0.34/2.56 ± 0.37	TG	Regular + ramipril
Ning Xiaoli [72]	2019	PNS	44/44	52/36	49.53 ± 7.11	3	—	TG	Hormone + LEFT
Xu Xiaoqin [73]	2020	PNS	35/35	45/25	43.21 ± 5.16/43.23 ± 5.15	3	5.08 ± 0.36/5.06 ± 0.35	TG	Prednisone
Jiang Liangyan [74]	2013	PNS	41/41	47/34	69.11 ± 5.51	9–12	1.34 ± 0.7	TG	Prednisone
Chen Weiwei [75]	2013	PNS	32/32	44/20	67.7 ± 5.6	12–18	0.4 ± 0.21	TG	Prednisone
Chen Yuanshu [76]	2018	PNS	38/38	44/32	48.87 ± 8.09/49.76 ± 8.21	3	2.41 ± 0.82/2.32 ± 0.76	TG	Prednisone acetate tablets + basic
Xu Junsan [77]	2017	PNS	48/46	49/45	73.47 ± 4.31/73.81 ± 4.58	12	—	TG	CTX
Li Weichao [78]	2013	PNS	15/15	17/13	29.3 ± 3.8	6	—	TG	Prednisone
Zhou Zhenzhong [79]	2015	PNS	20/20	23/17	65.23 ± 8.23/64.84 ± 7.42	4	0.79 ± 0.36/0.77 ± 0.44	TG	Prednisone
Li Xiaohong [80]	2015	PNS	40/40	47/33	53.5 ± 3.1/54 ± 3.3	—	1.6 ± 0.1/1.7 ± 0.2	TG	Prednisone + CTX
Liu Jinping [81]	2016	PNS	38/38	42/34	54.2 ± 3.7/54.1 ± 4.1	12	1.4 ± 1/1.5 ± 0.1	TG	Prednisone + CTX
Yang Yonglin [82]	2018	MN	47/44	52/39	66.62 ± 4.19/66.21 ± 3.98	6	—	TG	Benazepril
Qu Wei [83]	2016	MN	28/28	20/36	45.07 ± 11.93/38.82 ± 12.58	12	—	TG	Losartan potassium tablets
Mo Yaomei [84]	2019	MN	21/21	23/19	50.2 ± 9.08/49.8 ± 10.16	6	—	TG	Basics + MMF + prednisone
chen Ye [85]	2018	IgAN	25/24	33/16	9.3 ± 1.2/9.1 ± 1.1	3	—	Basic + prednisone + TG	Basic + prednisone

*Note. T* represents the experimental group and C represents the control group. *M* means male and F means female. “—” indicates that it is not reported or does not need to be reported. Drug dosage: glucocorticoid, 1 mg/(kg·d); (methyl)prednisone, 1–1.5 mg/kg·d; TG, 1–1.5 mg/kg·d; CTX, 8 mg/(kg·d); dipyridamole, 50 mg/time. Other drug dosages are not indicated in the literature.

**Table 2:** Results of meta-analysis of clinical efficacy.

Subgroup analysis	No. of studies	Weighted combination	Heterogeneity	Egger's bias *P* value	Metaregression *P* value
		OR [95% conf. interval]	*P* value			*I* ^2^	*P* value
*Disease type*							
NS	31	3.337 (2.649, 4.203)	*P* < 0.05	22.7%	*P* > 0.05	*P* < 0.05	*P* < 0.05
RNS	14	4.141 (3.037, 5.646)	*P* < 0.05	0.0%	*P* > 0.05	*P* < 0.05	
PNS	17	2.822 (2.109, 3.776)	*P* < 0.05	0.0%	*P* > 0.05	*P* > 0.05	
MN	3	4.761 (2.182, 10.385)	*P* < 0.05	0.0%	*P* > 0.05	*P* > 0.05	
IgAN	1	4.000 (1.129, 14.175)	*P* < 0.05	—	—	—	

*Mode of medication*							
Single drug	6	4.037 (2.241, 7.272)	*P* < 0.05	0.0%	*P* > 0.05	*P* < 0.05	*P* < 0.05
Combined use of drugs	60	3.373 (2.882, 3.947)	*P* < 0.05	0.0%	*P* > 0.05	*P* < 0.05	

*Course of treatment*							
<6 months	20	3.705 (2.854, 4.809)	*P* < 0.05	0.0%	*P* > 0.05	*P* < 0.05	*P* < 0.05
≥6 months	28	3.318 (2.622, 4.198)	*P* < 0.05	0.0%	*P* > 0.05	*P* < 0.05	
≥12 months	17	3.104 (2.272, 4.240)	*P* < 0.05	26.6%	*P* > 0.05	*P* > 0.05	

*Age of the patient (year)*							
0–18	2	4.981 (2.006, 12.365)	*P* < 0.05	0.0%	*P* > 0.05	—	*P* < 0.05
19–65	45	3.591 (2.974, 4.337)	*P* < 0.05	0.0%	*P* > 0.05	*P* < 0.05	
>66	18	3.454 (2.606, 4.577)	*P* < 0.05	0.0%	*P* > 0.05	*P* ≥ 0.05	
Not reported	1	0.167 (0.034, 0.805)	*P* < 0.05	—	—	—	—
**Total**	**66**	**3.415 (2.933, 3.975)**	*P* < 0.05	**0.0%**	*P* > 0.05	*P* < 0.05	—

**Table 3:** Results of meta-analysis of 24-hour urinary protein.

Subgroup analysis	No. of studies	Weighted combination		Heterogeneity		Egger's bias *P* value	Metaregression *P* value
		WMD [95% conf. interval]	*P* value	*I* ^2^	*P* value		
*Disease type*							
NS	25	−0.690 (−0.829, −0.551)	*P* < 0.05	94.2%	*P* < 0.05	*P* > 0.05	*P* > 0.05
RNS	12	−0.500 (−0.629, −0.370)	*P* < 0.05	77.7%	*P* < 0.05	*P* > 0.05	
PNS	13	−6.092 (−7.171, −5.014)	*P* < 0.05	99.8%	*P* < 0.05	*P* > 0.05	
MN	3	−0.290 (−0.533, −0.048)	*P* < 0.05	59.2%	*P* > 0.05	*P* > 0.05	
IgAN	1	−1.400 (−2.615, −0.185)	*P* < 0.05	—	—	—	

*Mode of medication*							
Single drug	5	−0.531 (−0.612, −0.451)	*P* < 0.05	85%	*P* < 0.05	*P* > 0.05	*P* > 0.05
Combined use of drugs	49	−0.501 (−0.525, −0.477)	*P* < 0.05	99.1%	*P* < 0.05	*P* < 0.05	

*Course of treatment*							
<6 months	17	−0.531 (−0.563, −0.498)	*P* < 0.05	99.7%	*P* < 0.05	*P* < 0.05	*P* > 0.05
≥6 months	22	−0.506 (−0.553, −0.459)	*P* < 0.05	94.9%	*P* < 0.05	*P* > 0.05	
≥12 months	14	−0.442 (−0.485, −0.399)	*P* < 0.05	74.1%	*P* < 0.05	*P* > 0.05	
Not reported	1	−1.720 (−2.140, −1.300)	*P* < 0.05	—	—	—	

*Age of the patient (year)*							
0–18	1	−0.657 (−0.766, −0.547)	*P* < 0.05	—	—	—	*P* > 0.05
19–65	39	−0.606 (−0.824, −0.388)	*P* < 0.05	93.4%	*P* < 0.05	—	
>66	14	−4.871 (−5.827, −3.915)	*P* < 0.05	99.7%	*P* < 0.05	—	
**Total**	**54**	**−0.503 (−0.526, −0.481)**	*P* < 0.05	**99.0%**	*P* < 0.05	*P* < 0.05	—

**Table 4:** Meta-analysis of reducing serum creatinine level in the experimental group and control group.

Subgroup analysis	No. of studies	Weighted combination		Heterogeneity	Egger's bias *P* value	Metaregression *P* value
		WMD [95% conf. interval]	*P* value	*I* ^2^			*P* value
*Disease type*							
NS	15	−11.764 (−14.967, −8.561)	*P* < 0.05	96.2%	*P* < 0.05	*P* < 0.05	*P* < 0.05
RNS	6	−6.479 (−8.960, −3.998)	*P* < 0.05	11.5%	*P* > 0.05	*P* > 0.05	
PNS	8	−11.101 (−13.966, −8.235)	*P* < 0.05	36.2%	*P* > 0.05	*P* > 0.05	
MN	2	2.304 (−20.951, 25.558)	*P* > 0.05	92.1%	*P* < 0.05	*P* > 0.05	
*Mode of medication*							
Single drug	1	−8.13 (−12.039, −7.233)	*P* < 0.05	0.0%	*P* > 0.05	—	*P* > 0.05
Combined use of drugs	30	−9.688 (−12.133, −7.243)	*P* < 0.05	94.4%	*P* < 0.05	*P* < 0.05	
*Course of treatment*							
<6 months	11	−12.520 (−16.473, −8.566)	*P* < 0.05	78.8%	*P* < 0.05	*P* > 0.05	*P* < 0.05
≥6 months	14	−6.177 (−8.431, −3.924)	*P* < 0.05	90.1%	*P* < 0.05	*P* < 0.05	
≥12 months	6	−3.029 (−9.316, 3.258)	*P* > 0.05	76.4%	*P* < 0.05	*P* > 0.05	
Not reported	1	−66.320 (−76.691, −55.949)	*P* < 0.05	—	—	—	
*Age of the patient (year)*							
19–65	21	−9.049 (−11.740, −6.357)	*P* < 0.05	94.7%	*P* < 0.05	*P* < 0.05	
>66	10	−11.120 (−13.762, −8.478)	*P* < 0.05	46.6%	*P* > 0.05	*P* > 0.05	
**Total**	**31**	**−9.636 (−12.039, −7.233)**	*P* < 0.05	**94.3%**	*P* < 0.05	*P* < 0.05	**—**

**Table 5:** Meta-analysis of reducing blood urea nitrogen level in the experimental group and control group.

Subgroup analysis	No. of studies	Weighted combination		Heterogeneity		Egger's bias *P* value	Metaregression *P* value
		WMD [95% conf. interval]	*P* value	*I* ^2^	*P* value		
*Disease type*							
NS	12	−0.536 (−0.832, −0.241)	*P* < 0.05	69.1%	*P* < 0.05	0.416	*P* > 0.05
RNS	6	−0.000 (−0.847, 0.846)	*P* > 0.05	97%	*P* < 0.05	0.252	
PNS	4	−0.114 (−1.360, 1.132)	*P* > 0.05	95%	*P* < 0.05	0.061	
MN	2	−0.555 (−1.204, 0.095)	*P* > 0.05	0.0%	0.673	—	
*Mode of medication*							
Single drug	1	−0.93 (−1.536, 0.324)	*P* > 0.05	84.3%	*P* < 0.05	—	*P* > 0.05
Combined use of drugs	23	−0.298 (−0.644, 0.049)	*P* > 0.05	91.6%	*P* < 0.05	*P* > 0.05	
*Course of treatment*							
<6 months	6	−0.38 (−1.07, 0.31)	*P* > 0.05	93.2%	*P* < 0.05	*P* > 0.05	*P* < 0.05
≥6 months	14	−0.235 (−0.693, 0.223)	*P* > 0.05	90.6%	*P* < 0.05	*P* > 0.05	
≥12 months	4	−0.484 (−0.859, −0.108)	*P* < 0.05	27.3%	*P* < 0.05	*P* > 0.05	
*Age of the patient (year)*							
19–65	17	−0.325 (−0.722, 0.071)	*P* > 0.05	92.2%	*P* < 0.05	*P* > 0.05	
>66	7	−0.310 (−1.046, 0.426)	*P* > 0.05	89.9%	*P* < 0.05	*P* > 0.05	
**Total**	**24**	**−0.326 (−0.661, 0.009)**	*P* > 0.05	**91.3%**	*P* < 0.05	*P* > 0.05	**—**

**Table 6:** Meta-analysis of elevated serum albumin levels in the experimental group and the control group.

Subgroup analysis	No. of studies	Weighted combination	Heterogeneity	Egger's bias *P* value	Metaregression *P* value
		WMD [95% conf. interval]	*P* value			*I* ^2^	*P* value
*Disease type*							
NS	20	4.407 (2.843, 5.971)	*P* < 0.05	92.3%	*P* < 0.05	*P* < 0.05	*P* < 0.05
RNS	12	5.726 (3.692, 7.760)	*P* < 0.05	95.1%	*P* < 0.05	*P* > 0.05	
PNS	10	3.796 (1.347, 6.245)	*P* < 0.05	94.7%	*P* < 0.05	*P* > 0.05	
MN	2	2.34 (-0.793, 5.483)	*P* > 0.05	38.2%	*P* < 0.05	*P* > 0.05	
IgAN	1	4.600 (0.484, 8.716)	*P* < 0.05	—	—	—	

*Mode of medication*							
Single drug	5	5.649 (4.712, 6.585)	*P* < 0.05	17.7%	*P* > 0.05	*P* > 0.05	*P* < 0.05
Combined use of drugs	40	4.448 (3.296, 5.600)	*P* < 0.05	93.9%	*P* < 0.05	*P* > 0.05	

*Course of treatment*							
<6 months	14	4.679 (3.050, 6.309)	*P* < 0.05	92.4%	*P* < 0.05	*P* > 0.05	*P* < 0.05
≥6 months	16	5.483 (3.455, 7.510)	*P* < 0.05	94.2%	*P* < 0.05	*P* > 0.05	
≥12 months	14	3.154 (1.468, 4.84)	*P* < 0.05	91.3%	*P* < 0.05	*P* > 0.05	
Not reported	1	7.920 (5.581, 10.259)	*P* < 0.05	—	—	—	

*Age of the patient (year)*							
0–18	1	4.600 (0.484, 8.716)	*P* < 0.05	—	*P* < 0.05	—	*P* < 0.05
19–65	35	4.606 (3.470, 5.743)	*P* < 0.05	91.3%	*P* < 0.05	*P* > 0.05	
>66	12	4.438 (2.110, 6.765)	*P* < 0.05	96.1%	*P* < 0.05	*P* > 0.05	
**Total**	**45**	**4.557** (**3.523**, **5.592**)	*P* < 0.05	**93.2%**	*P* < 0.05	*P* > 0.05	**—**

**Table 7:** Meta-analysis of the incidence of total adverse events.

Subgroup analysis	No. of studies	Weighted combination	Heterogeneity	Egger's bias *P* value	Metaregression *P* value
		OR [95% conf. interval]	*P* value			*I* ^2^	*P* value
*Disease type*							
NS	19	0.537 (0.399, 0.722)	*P* < 0.05	57.9%	*P* < 0.05	*P* < 0.05	*P* > 0.05
RNS	6	0.415 (0.248, 0.693)	*P* < 0.05	0.0%	*P* > 0.05	*P* > 0.05	
PNS	9	0.493 (0.330, 0.737)	*P* < 0.05	14.2%	*P* > 0.05	*P* > 0.05	
MN	1	1.412 (0.275, 7.257)	*P* > 0.05	—	—	—	
IgAN	1	3.556 (1.095, 11.546)	*P* < 0.05	—	—	—	

*Mode of medication*							
Single drug	4	0.458 (0.234, 0.899)	*P* < 0.05	41.2%	*P* > 0.05	*P* > 0.05	*P* > 0.05
Combined use of drugs	32	0.577 (0.465, 0.716)	*P* < 0.05	49.9%	*P* < 0.05	*P* < 0.05	

*Course of treatment*							
<6 months	9	0.616 (0.422, 0.900)	*P* < 0.05	61.1%	*P* < 0.05	*P* > 0.05	*P* < 0.05
≥6 months	19	0.580 (0.438, 0.769)	*P* < 0.05	51.4%	*P* < 0.05	*P* < 0.05	
≥12 months	7	0.293 (0.159, 0.540)	*P* < 0.05	0.0%	*P* > 0.05	*P* > 0.05	
Not reported	1	1.000 (0.219, 4.564)	*P* > 0.05	—	—	—	

*Age of the patient (year)*							
0–18	2	0.819 (0.371, 1.808)	*P* > 0.05	91.0%	*P* < 0.05	—	*P* < 0.05
19–65	25	0.541 (0.418, 0.701)	*P* < 0.05	44.5%	*P* > 0.05	*P* > 0.05	
>66	9	0.505 (0.340, 0.749)	*P* < 0.05	24.7%	*P* > 0.05	*P* > 0.05	
**Total**	**36**	**0.546** (**0.443**, **0.673**)	*P* < 0.05	**48%**	*P* < 0.05	*P* < 0.05	**—**

**Table 8:** Meta-analysis of the incidence of gastrointestinal adverse reactions.

Subgroup analysis	No. of studies	Weighted combination	Heterogeneity	Egger's bias *P* value	Metaregression *P* value
		OR [95% conf. interval]	*P* value			*I* ^2^	*P* value
*Disease type*							
NS	16	0.604 (0.391, 0.932)	*P* < 0.05	0.0%	*P* > 0.05	*P* > 0.05	*P* > 0.05
RNS	4	0.890 (0.414, 1.913)	*P* > 0.05	17.6%	*P* > 0.05	*P* > 0.05	
PNS	8	0.686 (0.340, 1.383)	*P* > 0.05	0.0%	*P* > 0.05	*P* > 0.05	
MN	2	1.859 (0.493, 7.002)	*P* > 0.05	0.0%	*P* > 0.05	*P* > 0.05	

*Mode of medication*							
Single drug	3	0.737 (0.258, 2.105)	*P* > 0.05	0.0%	0.455	*P* > 0.05	*P* > 0.05
Combined use of drugs	27	0.727 (0.523, 1.011)	*P* > 0.05	0.0%	0.73	*P* > 0.05	

*Course of treatment*							
<6 months	8	0.783 (0.428, 1.433)	*P* > 0.05	61.4%	*P* < 0.05	*P* > 0.05	*P* < 0.05
≥6 months	15	0.796 (0.520, 1.218)	*P* > 0.05	51.4%	*P* < 0.05	*P* > 0.05	
≥12 months	7	0.501 (0.236, 1.063)	*P* > 0.05	40.2%	*P* > 0.05	*P* > 0.05	

*Age of the patient (year)*							
0–18	1	2.211 (0.484, 10.092)	*P* > 0.05	91.0%	*P* < 0.05	—	*P* < 0.05
19–65	20	0.711 (0.482, 1.047)	*P* > 0.05	47.7%	*P* < 0.05	*P* > 0.05	
>66	9	0.642 (0.356, 1.159)	*P* > 0.05	24.7%	*P* > 0.05	*P* > 0.05	
**Total**	**30**	0.711 (0.517, 0.980)	*P* < 0.05	**0.0%**	*P* > 0.05	*P* < 0.05	**—**

## Data Availability

The data used to support the findings of this study are included within the article.
